# Dendrobium nobile Lindley and its bibenzyl component moscatilin are able to protect retinal cells from ischemia/hypoxia by dowregulating placental growth factor and upregulating Norrie disease protein

**DOI:** 10.1186/s12906-018-2256-z

**Published:** 2018-06-22

**Authors:** Wen-Haur Chao, Ming-Yi Lai, Hwai-Tzong Pan, Huei-Wen Shiu, Mi-Mi Chen, Hsiao-Ming Chao

**Affiliations:** 1 Institute of Pharmacology, School of Medicine, National Yang-Ming University, Taipei, Taiwan; 2 Department of Ophthalmology, Cheng Hsin General Hospital, Taipei, Taiwan; 3 Department of Ophthalmology, Taipei Medical University-Shuang Ho Hospital, New Taipei City, Taiwan; 4 Department of Chinese Medicine, School of Chinese Medicine, China Medical University, Taichung, Taiwan

**Keywords:** Dendrobium nobile Lindley, Moscatilin, Retinal ischemia, Oxygen glucose deprivation, Placental growth factor, Norrie disease protein

## Abstract

**Background:**

Presumably, progression of developmental retinal vascular disorders is mainly driven by persistent ischemia/hypoxia. An investigation into vision-threatening retinal ischemia remains important. Our aim was to evaluate, in relation to retinal ischemia, protective effects and mechanisms of Dendrobium nobile Lindley (DNL) and its bibenzyl component moscatilin. The therapeutic mechanisms included evaluations of levels of placental growth factor (PLGF) and Norrie disease protein (NDP).

**Methods:**

An oxygen glucose deprivation (OGD) model involved cells cultured in DMEM containing 1% O_2_, 94% N_2_ and 0 g/L glucose. High intraocular pressure (HIOP)-induced retinal ischemia was created by increasing IOP to 120 mmHg for 60 min in Wistar rats. The methods included electroretinogram (ERG), histopathology, MTT assay and biochemistry.

**Results:**

When compared with cells cultured in DMEM containing DMSO (DMSO+DMEM), cells subjected to OGD and pre-administrated with DMSO (DMSO+OGD) showed a significant reduction in the cell viability and NDP expression. Moreover, cells that received OGD and 1 h pre-administration of 0.1 μM moscatilin (Pre-OGD Mos 0.1 μM) showed a significant counteraction of the OGD-induced decreased cell viability. Furthermore, compared with the DMSO+OGD group (44.54 ± 3.15%), there was significant elevated NDP levels in the Pre-OGD Mos 0.1 μM group (108.38 ± 29.33%). Additionally, there were significant ischemic alterations, namely reduced ERG b-wave, less numerous retinal ganglion cells, decreased inner retinal thickness, and reduced/enhanced amacrine’s ChAT/Müller’s GFAP or vimentin immunolabelings. Moreover, there were significantly increased protein levels of HIF-1α, VEGF, PKM2, RBP2 and, particularly, PLGF (pg/ml; Sham vs. Vehicle: 15.11 ± 1.58 vs. 39.53 ± 5.25). These ischemic effects were significantly altered when 1.0 g/Kg/day DNL (DNL1.0 + I/R or I/R+ DNL1.0) was applied before and/or after ischemia, but not vehicle (Vehicle+I/R). Of novelty and significance, the DNL1.0 action mechanism appears to be similar to that of the anti-PLGF Eylea [PLGF (pg/ml); DNL1.0 vs. Eylea+I/R: 19.93 ± 2.24 vs. 6.44 ± 0.60].

**Conclusions:**

DNL and moscatilin are able to protect against retinal ischemic/hypoxic changes respectively by downregulating PLGF and upregulating NDP. Progression of developmental retinal vascular disorders such as Norrie disease due to persistent ischemia/hypoxia might be thus prevented.

**Electronic supplementary material:**

The online version of this article (10.1186/s12906-018-2256-z) contains supplementary material, which is available to authorized users.

## Background

Defects in vasculogenesis (early retinal vessel development) seem to be mediated through the Norrin-dependent Wnt signaling pathway [1–3]. Norrin/Frizzled-4 signaling seems to play a crucial role in vasculogenesis such as in Norrie disease and familial exudative vitreoretinopathy [1–4], which might eventually progress into retinal ischemia and neovascularization (NV; angiogenesis). In addition to Norrie disease and familial exudative vitreoretinopathy, there were other developmental retinal vascular disorders, namely Coats disease and persistent hyperplastic primary vitreous that share similar fundus pictures, namely peripheral retinal avascularization and subretinal exudation [1–5]. As above mentioned, these vitreoretinopathies may also cause retinal ischemia, thus giving rise to a similar threat to the patient’s vision, although they are not as common as other retinal ischemic disorders such as central/branch retinal artery occlusion (CRAO/BRAO), central/branch retinal vein occlusion (CRVO/BRVO), glaucoma, diabetic retinopathy (DR) and neovascular age related macular degeneration (nvAMD) [6]. As indicated by Beck et al. (2017) [7], persistent hypoxia has been assumed to be one of the major driving forces involved in progression of these developmental retinal vascular disorders such as Norrie disease [5, 7]. Based on the role of Norrin in vasculogenesis [1–5, 7], we investigated here whether the level of Norrie disease protein (NDP; Norrin) might change in cells subjected to a hypoxia model system (oxygen glucose deprivation, OGD). We also investigated whether moscatilin, the bibenzyl component of Dendrobium nobile Lindley (DNL), might have the capacity to upregulate the expression level of NDP, which could potentially protect retinal cells from ischemia/hypoxia that seems to induce the progression of these developmental retinal vascular disorders.

Retinal ganglion cells (RGCs) and amacrine cells in the inner retina are susceptible to ischemia/reperfusion (I/R) [8]. Moreover, vimentin/glial fibrillary acidic protein (GFAP) immunolabelling of Müllers is elevated after ischemia [9]; this is also associated with a reduction in RGC numbers [8]. Overexpression of vascular endothelium growth factor (VEGF), of hypoxia inducible factor (HIF-1α), of pyruvate kinase M2 (PKM2) and of retinoblastoma-binding protein 2 (RBP2) are also known to occur together in the ischemic retina [8, 10–12] and further abnormal NV (late neovessel formation) can lead to visual dysfunction due to edema and hemorrhage. Upregulation of HIF-1α and VEGF has also be observed in the Norrin depleted retina [5]. In addition to VEGF [8, 10, 12], placental growth factor (PLGF) has been reported to be increased when there are defined ischemic disorders of the retina/choroid vasculature; thus, downregulation of this factor is able to be utilized as a biomarker for visual functional outcome and treatment [13]. The present study aims to provide further confirmation regarding these ischemic alterations.

As described in “*An Illustrated Chinese Materia Medica*”, DNL (a member of the Orchidae family) is a “vision improving” herbal. DNL has also been used as a tonic and found to have antipyretic/anti-inflammatory effects [14] and anti-angiogenic (e.g. anti-VEGF/HIF-1α) properties [15–17]. DNL has several active ingredients with various action mechanisms, including alkaloids (Tissue necrosis factor receptor 1 protein overexpression via inhibiting the p-p38 mitogen activated protein kinase and NF-ĸB pathway) [18], flavonal glycosides (α-glucosidase inhibitors) [19], SG-168 and polysaccharides (antioxidative effects) [20, 21]. Furthermore, an anti-angiogenic or anti-oxidative compound moscatilin is one of the active bibenzyl compounds present in DNL and this chemical seems to have a range of effects. These include an anti-VEGF/HIF-1α effect, where it acts as. OH radical scavenger, an anti-inflammation effect and an anti-apoptosis effect [15–17, 22, 23]; there are also other unknown mechanisms of action that are different to those mentioned above. In other words, DNL could possess a number of distinct therapeutic effects that may be shared with its component moscatilin.

The aim of the present study is to examine whether DNL is able to attenuate retinal ischemic injury (see also *Ischemia induction* in the Methods) in the rat. Additionally, the effects of DNL and its mechanisms of action were assessed by electrophysiology, by examining the thickness of various retinal layers, by assessing RGC number, and by examining choline acetyl transferase (ChAT) immuolabeling in amacrine cells and by observing vimentin/GFAP immunoreactivity in Müller cells. Moreover, protein expression levels of HIF-1α, VEGF, PKM2 and RBP2 were analyzed. Furthermore, we for the first time investigated under hypoxia/ischemia the effect of DNL and, of novelty and significance, of its bibenzyl component moscatilin (0.1 μM; non-toxic at the concentration≤1 μM) [23] on vasculogenesis/angiogenesis in relation to the expression levels of NDP (norrin)/PLGF. As part of the NDP study, a RGC-5 cell system (retinal neuronal progenitors; see also in vitro studies in the Methods) model, namely OGD, was used to investigate the mechanisms involved in hypoxic/ischemic-like injury; this part of the study involved the assessment of 3-(4,5-dimethylthiazol-2-yl)-2,5-diphenyl-2H-tetrazolium bromide (MTT) cell viability and the measurement of the expression levels of NDP by the Western blotting assay.

## Methods

### Chemicals

DNL was purchased from the Ko Da Company (Taipei, Taiwan) and dissolved in ddH_2_O. Moscatilin was purchased from EMMX Biotechnology (EN10271, CA, USA) and dissolved in dimethyl sulfoxide (DMSO; vehicle). Various inhibitors/antibodies were purchased from various companies, namely JIB-04 (Sigma-Aldrich), Shikonin (S7576; Sigma-Aldrich), Avastin (Hoffmann-La Roche), or Eylea (Regeneron Pharmaceuticals Inc.).

### In vitro studies

#### Oxygen glucose deprivation and cell treatment

The RGC-5 cells are not transformed rat RGCs but, rather, mouse retinal neuronal precursors [24]. OGD [25] was defined as cells that were maintained in glucose-free Dulbecco’s modified Eagle medium (DMEM; Thermo Fisher Scientific Inc.) at 37 °C under the hypoxic (ischemic-like) conditions, namely 1% O_2_ (monitored by an analyzer; a Penguin Incubator: control range 1~ 89%; Astec Company, Kukuoka, Japan), 94% N_2_ and 5% CO_2_. There were different groups (Table 1), consisting of cells that received (i) DMSO in DMEM (control cells; DMSO+DMEM), (ii) DMSO followed by OGD (DMSO+OGD), (iii) OGD and administration of moscatilin (0.1 μM in DMEM) at 1 h pre-OGD (Pre-OGD Mos 0.1 μM), (iv) during OGD (During OGD Mos 0.1 μM), or (v) at 1 h post-OGD (Post-OGD Mos 0.1 μM). At the end of the 1 day OGD period, the cell cultures were returned to fresh DMEM for another 24 h. The MTT (viability) and the Western blotting assays (NDP) were then performed.

**Table 1:** Group names and definition of the conditions used to treat the various experimental groups of cells or animals. In vitro MTT/Western blotting experiments^a^

Group names	Definition of conditions of cells that received
DMSO+DMEM (control)	DMSO in DMEM
DMSO+OGD	OGD and treatment with DMSO at 1 h pre-OGD
Pre-OGD Mos 0.1 μM	OGD and treatment with moscatilin at 1 h pre-OGD
During OGD Mos 0.1 μM	OGD and treatment with moscatilin during OGD
Post-OGD Mos 0.1 μM	OGD and treatment with moscatilin at 1 h post-OGD

^a^The number of experiments in MTT assay for cell viability (*n* = 6) and Western blot assay for NDP (*n* = 3). The test compounds were persistently included in the culture medium during the OGD period as defined. Moscatilin (0.1 μM) was kept in DMEM from the administration. *Abbreviations*: *NDP* Norrie disease protein, *DMSO* dimethyl sulfoxide, *OGD* oxygen glucose deprivation

### MTT cell viability assay

Mitochondria nicotinamide adenine dinucleotide phosphate (NADPH) dependent oxidoreductases are capable of reducing MTT to form formazan [26]. Therefore, an increase in the amount of dark purple formazan corresponds to greater cell viability. MTT (0.5 mg/mL; Sigma-Aldrich) was added for 3 h at 37 °C to the 96-well plates containing the original 100 μL of cells. The reduced MTT was then solubilized by adding 100 μL DMSO. After agitation of the plates, the optical density (OD) of the solubilized formazan was measured using an ELISA reader (Synergy H1 Multi-Mode Reader BioTek Instruments) at 562 nm. Cell viability is expressed as OD values relative to the control (100%).

### In vivo studies

#### Animals

The animal use protocol has been reviewed and approved by the Institutional Animal Care and Use Committee at Cheng Hsin General Hospital (CHGH; Taipei, Taiwan; Approval No: CHIACUC 104–14). A large plastic cage (Shineteh Instruments Co., Ltd., Taipei) was used to keep at most six six-week-old Wistar rats (250–300 g; BioLasco, Taipei) at a humidity of 40 to 60% and a temperature of 19 to 23 °C. For the electroretinogram (ERG) and histopathology [cresyl violet, choline acetyl transferase (ChAT) and vimentin/GFAP] studies, the animals were randomly distributed into various groups (Table 2), i.e. Sham (*n* = 12), Vehicle+I/R (n = 12), DNL0.5 + I/R (n = 12), DNL1.0 + I/R (n = 12), I/R + Vehicle (*n* = 10), and I/R + DNL1.0 (*n* = 10). In addition, for the Western blotting/ELISA assays, the rats were randomly distributed into following groups (Table 3), namely Sham (*n* = 10), Vehicle+I/R (*n* = 10), DNL1.0 + I/R (*n* = 10), and I/R plus pre-ischemia i.v.i. substances [JIB-04 (*n* = 4); Shikonin (*n* = 7); Avastin (*n* = 4); Eylea (*n* = 4)]. The number of animals utilized for various defined procedures was 120 (=68 + 49 + 3 = 117 + 3) and this included animals (*n* = 3) that died during the retinal ischemia. Additionally (*n* = 16), during the fluorogold retrograde labeling for RGCs, a comparison was made between various groups (Table 4), namely Sham (*n* = 4), Vehicle+I/R (*n* = 4), DNL1.0 + I/R (n = 4), I/R + DNL1.0 (n = 4). In total, the overall number of the animals used was 136 (=120 + 16; Tables 2, 3 and 4). All animals were kept on a 12-h light/dark cycle with 12–15 air changes/hour. The animals were provided with food and water at liberty.

**Table 2:** Group names and definition of the conditions used to treat the various experimental groups of cells or animals. In vivo eletrophysiological and histopathological experiments^a^

Group names	Definition of conditions of animals that received
i. Sham (*n* = 12; control)	sham procedure
ii. Vehicle+I/R (*n* = 12)	Pre-ischemic treatment with vehicle followed by I/R
iii. DNL0.5 + I/R (*n* = 12)	Pre-ischemic treatment with DNL0.5 followed by I/R
iv. DNL1.0 + I/R (*n* = 12)	Pre-ischemic treatment with DNL1.0 followed by I/R
v. I/R + Vehicle (*n* = 10)	I/R followed by post-ischemic treatment with vehicle
vi. I/R + DNL1.0 (*n* = 10)	I/R followed by post-ischemic treatment with DNL1.0

^a^These animals were evaluated by ERG and sacrificed for histopathological studies, namely cresyl violet, ChAT and vimentin/GFAP labelings. Subtotally, 68 animals were used. Pre−/post-ischemia oral gavage of 0.5 g/kg/day (DNL0.5 + I/R), 1.0 g/kg/day of DNL (DNL1.0 + I/R; I/R + DNL1.0), or the same volume of vehicle was given (Vehicle+I/R; I/R + Vehicle). *Abbreviations*: *ChAT* choline acetyltransferase, *GFAP* glial fibrillary acidic protein

**Table 3:** Group names and definition of the conditions used to treat the various experimental groups of cells or animals. In vivo Western blotting/ELISA experiment^a^

Group names	Definition of conditions of animals that received
i. Sham (*n* = 10; control)	sham procedure
ii. Vehicle+I/R (*n* = 10)	Pre-ischemic oral vehicle followed by I/R
iii. DNL1.0 + I/R (*n* = 10)	Pre-ischemic oral DNL1.0 followed by I/R
iv. JIB-04 + I/R (*n* = 4)	Pre-ischemic intravitreous JIB-04 followed by I/R
v. Shikonin+I/R (*n* = 7)	Pre-ischemic intravitreous shikonin followed by I/R
vi. Avastin+I/R (*n* = 4)	Pre-ischemic intravitreous avastin followed by I/R
vii. Eylea+I/R (*n* = 4)	Pre-ischemic intravitreous eylea followed by I/R

^a^These animals were evaluated by Western blotting/ELISA assays and sacrificed for the measurement of various proteins, namely HIF-1α, VEGF, PKM2, RBP2 and PLGF. Subtotally, 49 animals were used. The number of animals utilized for various defined procedures was 120 (=68 + 49 + 3) and this included animals (*n* = 3) that died during the retinal ischemia. Pre-ischemia intravitreous injection of 1.0 g/kg/day of DNL (DNL1.0 + I/R), or inhibitors/antibodies of PKM2 (shikonin), RBP2 (JIB-04), VEGF-A (avastin), PLGF (Eylea), or the same volume of vehicle was administered. *Abbreviations*: *HIF-1α* hypoxia inducible factor, *VEGF* vascular endothelium growth factor, *PKM2* pyruvate kinase M2, *RBP2* retinoblastoma-binding protein 2, *PLGF* placental growth factor

**Table 4:** Group names and definition of the conditions used to treat the various experimental groups of cells or animals. In vivo fluorogold RGC experiment^a^

Group names	Definition of conditions of animals that received
i. Sham (*n* = 4; control)	sham procedure
ii. Vehicle+I/R (*n* = 4)	Pre-ischemic treatment with vehicle followed by I/R
iii. DNL1.0 + I/R (*n* = 4)	Pre-ischemic treatment with DNL1.0 followed by I/R
iv. I/R + DNL1.0 (*n* = 4)	I/R followed by post-ischemic treatment with DNL1.0

^a^These animals were evaluated by the fluorogold retrograde labeling for RGC. Subtotally, 16 animals were used. In total, the overall number of the animals used was 136 (=120 + 16). Pre−/post-ischemia oral gavage of 1.0 g/kg/day of DNL (DNL1.0 + I/R; I/R + DNL1.0), or the same volume of vehicle was applied

### Drug administration

For the electrophysiological, immuohistochemical and molecular biological studies, drug administration was carried out for seven days and involved a number of different groups (Tables 1, 2, 3 and 4), namely a post-ischemic administration (daily high dose of DNL at 1 g/kg/day, I/R + DNL1.0), or pre-ischemic administration (daily high dose of DNL, DNL1.0 + I/R; low dose of DNL at 0.5 g/kg/day, DNL0.5 + I/R) [21]. The rats in the vehicle group that were subjected to ischemia were either post-administrated (I/R + Vehicle) or pre-administrated with a similar volume of vehicle (Vehicle+I/R) as the DNL group. One/seven days after retinal ischemia and pre-ischemic/post-ischemic administration of defined compounds, the rats were sacrificed; this was also done either one or seven days after sham procedure. To reduce the number of the animals used, in these cases only a high dose of DNL (I/R + DNL1.0) were administered after ischemia.

As compared with the Sham, Vehicle+I/R or DNL1.0 + I/R group, the ischemic eyes of other groups (Tables 1, 2, 3 and 4) received intravitreal injections using a 30-gauge needle connected to a 25 μl syringe after the pupil was dilated with 1% tropicamide and 2.5% phenylephrine. Specifically, the ischemic eyes received 1 day pre-ischemia intravitreal administrations of one of the various inhibitors/antibodies, namely 10 μM/5 μl JIB-04, 4 μM/5 μl Shikonin, 125 μg/5 μl Avastin, or 200 μg/5 μl Eylea. One day after retinal I/R and administration of relevant compounds or after a sham procedure, the animals were sacrificed to minimize degradation of the proteins of interest, for example, the occurrence of degradation 7 days after retinal ischemia. Retinal samples were used to measure the protein levels of HIF-1α, RBP2, PKM2, VEGF-A and PLGF by the Western blot analysis or ELISA. Rats were also sacrificed in order to allow analysis of the retina by various methods such as ERG and cresyl violet, flurogold, ChAT, vimentin or GFAP staining histopathology. This was carried out one day after retinal ischemia and pre-ischemic administration of various compounds. On the other hand, the post-ischemia treatment groups were followed up for 7 days in order to observe any long-term (chronic) post-ischemic alterations.

In previous reports, higher concentrations of moscatilin (1.25~ 20 μM) have been shown to be able to dose-dependently and time-dependently reduce cell viability with IC_50_ at 7.0 and 6.7 μM for 24 h in two cell lines, respectively [17]; thus, 0.1 μM, which is a non-toxic concentration [23], was presently used to evaluate the compound’s protective effect against OGD. Moscatilin (0.1 μM) was administered at 1 h pre-OGD, during OGD, or alternatively at 1 h post-OGD (Tables 1, 2, 3 and 4). The therapeutic effects of the drug were evaluated by MTT and Western blotting analysis.

### Establishing retinal ischemia

#### Anesthesia and euthanasia

An intraperitoneal injection (i.p.) of 100 mg/kg ketamine (Pfizer) and 5 mg/kg xylazine (Sigma-Aldrich) was used to anesthetize the animals. Furthermore, at least 140 mg/kg sodium pentobarbital (SCI Pharmtech) was intraperitoneally given to humanely kill the animals (Scientific Procedures Acts 1986).

#### Ischemia induction

Each rat was anesthetized with the above anesthetics and placed in a stereotaxic frame. The anterior chamber of one eye was cannulated using a 30-gauge needle linked to an elevated 0.9% saline reservoir; this was used to bring about an increase in intraocular pressure (IOP) to 120 mmHg for 1 h [8]. A whitening of the retina indicated a build-up of ischemic injury. A sham version of the above-mentioned ischemia induction procedure, but without the elevation of the saline bottle connected to the rat’s eye, was carried out as a control. Animals were placed on a heating pad at 37°C and kept normothermic during ischemia & the following 3-h reperfusion.

### Flash ERG measurements

Flash ERG were recorded from all the animals before the sham procedure or I/R (day 0), and one day after the sham procedure or I/R with pre-administration of the various drugs. In the post-administration group, ERG data were recorded for all the animals pre-ischemia (day 0), and post-ischemia (day 1, 3, 5 or 7 after ischemia and administration of appropriate compounds). Dark adaption was allowed for 8 h and then anesthesia was carried out to allow recording of the ERG after dilation of pupils. A stimulus of 0.5 Hz was provided using a strobe 2 cm before the animal’s eye. Fifteen continuous recordings were collected at two-second interval and at 10 kHz; their amplitudes were maximized and calculated to obtain an average; this involved the use of an amplifier (P511), a regulated power supply (RPS 107) and a stimulator (PS22), all obtained from Grass-Telefactor. To allow comparisons between the various groups, the ratio of the b-wave amplitude of one eye (sham or ischemia) to that of the untreated fellow normal eye was measured [8].

### Cresyl violet staining

Across all groups, after the rats were sacrificed, they received an intracardial perfusion of physiologic saline. The eyeballs were marked at the 12 o’clock on the cornea using a silk suture and then enucleation was carried out. This was followed by fixation in 4% paraformaldehyde at 4 °C for 24 h, dehydration in a graded ethanol series and embedding in paraffin (Tissue-Tek TEC 5; Sakura). Sectioned samples (5 μm) were obtained along the vertical meridian. These were subjected to cresyl violet labeling and were then observed under a light microscope (Leica). Each retinal section was photographed at the same magnifying power and the retinal thickness of the various different layers was measured from photographs (Ilford Pan-F plus film, 50 ASA). To quantify the degree of retinal ischemic injury, firstly, the whole retinal thicknesses was measured from the internal limiting membrane (ILM) to retinal pigment epithelium (RPE) layer. Secondly, the inner retinal thickness from the ILM to inner nuclear layer (INL) were measured by an expert who was masked to the conditions under which the sectioned samples had been obtained. The various experimental groups were compared to the control group (sham).

### RGC retrograde staining

Under anesthesia, a 2-cm incision was created in the animal’s scalp and two small holes were drilled into the skull as illustrated [8]. Next, injections of 10-μl of 5% fluorogold (Sigma-Aldrich) were carried out using a micropipette at 3.8, 4.0, and 4.2 mm below the surface. The fluorogold was injected 3 days before the animals were sacrificed. Retrieval, fixation, dissection and processing of retinal samples were performed as described previously [8]. The RGC density was calculated as the ratio of the total number of RGCs divided by the total area of the retinal sample [8].

### Immunofluorescence analysis

After sacrifice of the animals and intracardial perfusion, the eyeballs of the rats were enucleated, fixed for 45 min, dehydrated and finally embedded in paraffin as above-mentioned. Sampling was carried out 1 day after the sham procedure or induction of retinal ischemia with pre-ischemia/post-ischemia administration of DNL or vehicle. Each 5 μm retinal section was incubated overnight with primary antibodies, namely either goat anti-ChAT polyclonal antibody (1:100; AB144p; Chemicon), mouse anti-vimentin monoclonal antibody (1:100; V6630; Sigma-Aldrich), or rabbit anti-GFAP polyclonal antibody (Millipore). Next, retinal sections were incubated with an appropriate secondary antibody, either rhodamine-conjugated rabbit anti-goat antibody (1:500; AP106R; Chemicon), or fluorescein isothiocyanate (FITC)-conjugated goat anti-mouse IgG (1:500; AP124F; Millipore)/anti-rabbit IgG (AP132F; 1:500; Millipore). In parallel, cellular nuclei were labeled using 4′,6-diamidino-2-phenylindole (DAPI; Molecular Probes). Finally, retinal sections were examined using a fluorescence microscope (Olympus BX61) by an expert who was masked to the conditions under which the sectioned samples had been obtained in order to grade the immunolabeling level of various different groups against the control group (sham).

### Western blotting assays

Retinal samples or samples of cells were sonicated in lysis buffer, namely mammalian protein extraction reagent (MPER; HyCell). Identical quantities of denatured proteins (40 μg/30 μl/well) then underwent sodium dodecyl sulfate polyacrylamide gel electrophoresis (SDS-PAGE; Bio-Rad) as described previously [10]. After separation, the protein bands were transferred to a polyvinylidene difluoride membrane, which was treated for 12 h at 4 °C with the following primary antibodies, mouse monoclonal anti-β-actin antibody (AC-15; 1:2000; ab6276)/anti-HIF-1α antibody (1:200; H1alpha67-ChIP Grade; Abcam Inc.), rabbit polyclonal anti-VEGF antibody (A-20; 1:200; sc-152)/anti-PKM2 antibody (1:500; ab38237), or rabbit monoclonal anti-RBP2 antibody (ab177486; 1:1000; Abcam Inc.). The blots were next treated with relevant secondary antibody, HRP-conjugated goat anti-rabbit IgG (1:5000; Santa Cruz Biotechnology Inc.) or goat anti-mouse IgG (1:5000; sc-2005) at 37 °C for 1 h. Dilution of primary/secondary antibodies was carried out in 5% fat-free skimmed milk. Finally, the membranes were processed using an enhanced chemiluminescent analysis system (HyCell) and exposed to an X-ray film (Fujifilm). The amount of each protein was then evaluated by scanning densitometry.

### Enzyme-linked immunosorbent assay

The level of PLGF was determined using ELISA [27]. One day after ischemia, the retina was separated from the enucleated eye cup, dissociated and then lysed by incubation in MPER (Hycell) for 30 min; the lysate was then centrifuged at 13,000 rpm for another 30 min. The total protein in each sample was determined using a bicinchoninic acid protein kit (Thermo Fisher Scientific) [28]. The PLGF levels in the supernatant were measured using a PLGF ELISA kit (CSB-E07400r; Cusabio Life Scince) accordingly. Anti-PLGF antibody had been previously coated onto the microwell. After twice washing each well with 200 μL wash buffer over 15 min, the PLGF in the samples or various concentrations of PLGF standard protein (100 μL) was allowed to bind to the antibodies coating the microwells at room temperature for 2 h on a shaker (75 rpm). After washing each well twice with 200 μL wash buffer [Phosphate buffered saline with Tween-20 (PBST)], 100 μL of biotin-conjugated anti-PLGF antibody (diluted in assay buffer: PBST and 0.5% BSA) was added to each well for 1 h on a shaker (75 rpm), which allowed the binding to the PLGF captured by the coated antibody. After twice washing to remove unbound biotin-conjugated anti-PLGF, 100 μL avidin- horseradish peroxidase (HRP; diluted in assay buffer) was added; this bound to the biotin conjugated anti-VEGF-A antibody (75 rpm). Following 1-h incubation on a shaker, the unbound avidin-HRP was removed by washing twice. Finally, 90 μL of 3,3′,5,5′-trimethylbenzene (TMB; 100 μM) solution, which is oxidized by HRP, was added to each well for 20 min. The color was then changed to yellow and the reaction was stopped by the addition of 50 μL stop solution (sulfuric acid solution, 100 μM). The maximum absorbance (OD) at 450 nm was detected immediately using a microplate reader (Synergy H1 Hybrid Multi-Mode Reader, Biotek ELx800). The PLGF concentration of each sample was determined by constructing a standard curve using various amounts of PLGF (0, 3.125, 6.25, 12.5, 25, 50, 100 and 200 pg/mL). The instrument was adjusted to zero using a 100 μL sample diluent, which served as the blank. The results are expressed as ODs relative to that of the control group (100%).

### Statistical analysis

Comparisons between two groups were made using the unpaired Student’s *t*-tests. One-way analysis of variance (ANOVA) was performed to compare three or more independent groups. Following the one-way ANOVA, the Dunnet’s test was used to compare the control (e.g. Vehicle+I/R) with all other groups (e.g. DNL1.0 + I/R). All results were shown as means±SE. A value of *P* < 0.05 was considered significant.

## Results

### MTT cell viability assay

**Fig. 1:**
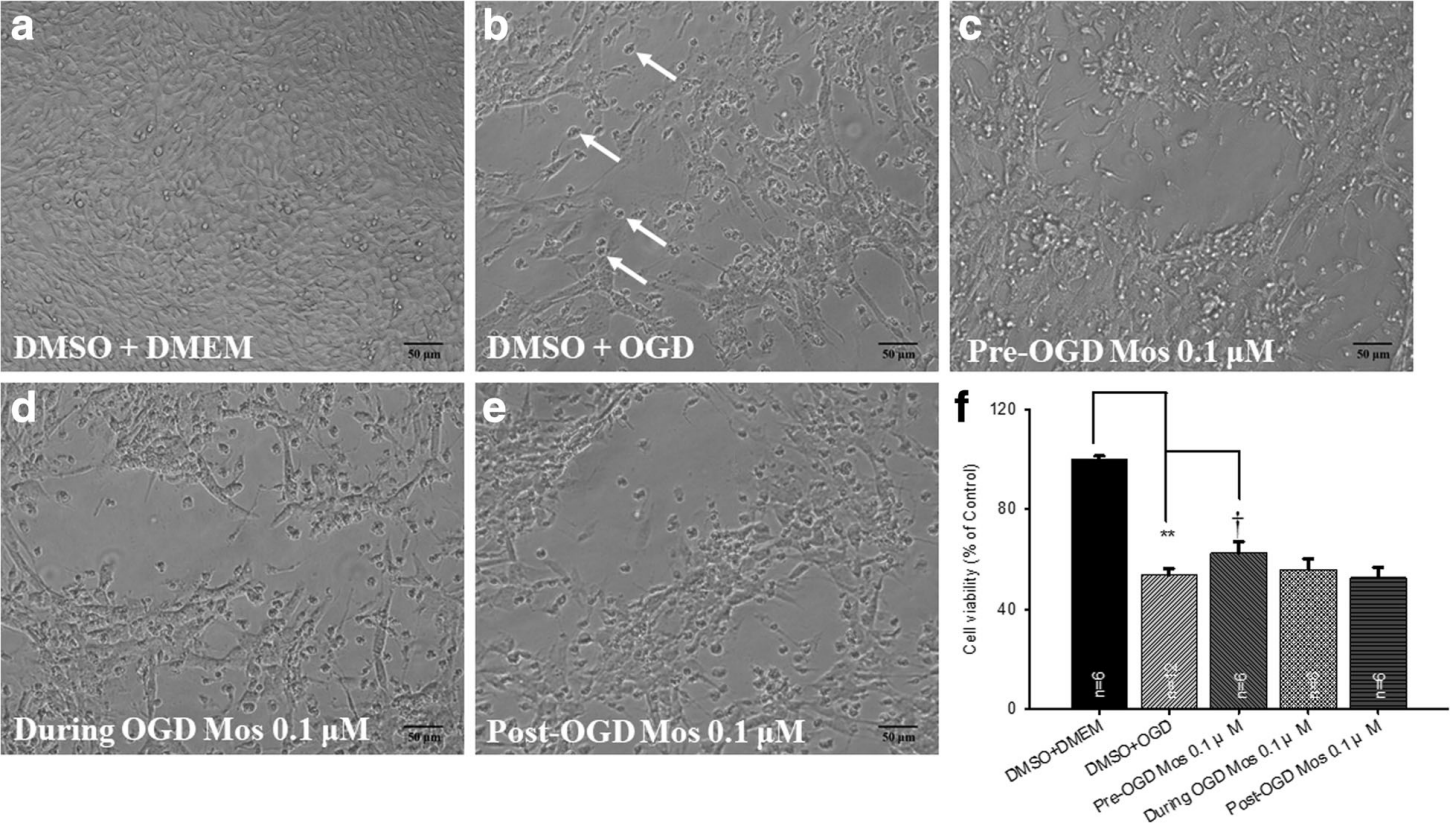
**a**~**e** The cell viability study of the effects of moscatilin on RGC-5 cells subjected to OGD using light microscopy. After pre-administration of DMSO (vehicle) followed by OGD (DMSO+OGD), the cells were found to be less numerous and some were deformed (as indicated by arrows) as compared to the normal control group (cells cultured in DMEM and pre-administration of DMSO; DMSO+DMEM). These OGD-induced alterations were mitigated by pre-administration of moscatilin (Mos) at 1 h pre-OGD (Pre-OGD 0.1 μM Mos). **f** The effects of moscatilin on cells subjected to OGD analyzed quantitatively by MTT assay. ** indicates a significant difference (*P* < 0.01) between the control (DMSO+DMEM) and the “DMSO+OGD” group. † indicates a significant difference (*P* = 0.04) between the “DMSO+OGD” group and the “Pre-OGD 0.1 μM Mos” group. Please refer to Table 1 for definitions of the other two groups, namely “During OGD 0.1 μM Mos” and “Post-OGD 0.1 μM Mos”. Results are presented as means±S.E.M. (*n* = 6). DMSO, dimethyl sulfoxide; DMEM, Dulbecco’s modified Eagle’s medium; OGD, oxygen glucose deprivation; RGC, retinal ganglion cell; MTT, 3-(4,5-dimethylthiazol-2-yl)-2,5-diphenyltetrazolium bromide. Scale = 50 μm

Firstly, changes in the cell morphology and the number of RGC-5 cells were examined by light microscopy. The cells cultured in the DMEM with pre-administration of DMSO (DMSO+DMEM; Fig. 1a) had a pyramidal shape and exhibited a characteristic neuronal morphology. By way of contrast to the DMSO+DMEM group, the cells subjected to OGD and pre-administrated with DMSO (DMSO+OGD) were deformed (as indicated by white arrows; Fig. 1b); moreover, there was also a considerable reduction in their cell number. Next, the effects of administering moscatilin (0.1 μM to 100 μM) to cells subjected to OGD were observed. Specifically, the protective effect of pre-OGD administration of moscatilin on the OGD was demonstrated by increased cell viabilities at 0.1 (62.65 ± 4.35%; *n* = 6) or 1 μM (66.36±9.35%; *n* = 4); however, a cytotoxic effect, namely a decrease in the cell viability, was shown at 10 (30.60 ± 9.05%; *n* = 4) and 100 μM (12.75 ± 3.34%; n = 4). The latter two concentrations would seem to be beyond the pharmacological protective levels. This is not inconsistent with previous research, which has indicated that moscatilin acts in a time-dependent (1~ 3 days) and dose-dependent manner (1.25~ 20 μM) [17]. The compound seems to have a cytotoxic effect that is probably related to its ability to induce a G2 phase arrest in the mitosis at the concentration of 20 or 50 μM as early as 15 h posttreatment [29]. However, the concentrations of moscatilin equal to or less than 1 μM have been proved to be non-toxic and are able to act as a potent. OH radical scavenger [23]. An investigation into how a low concentration of moscatilin (0.1 μM) is able to increase the cell viability was then carried out. As compared with the DMSO+OGD group (Fig. 1b), administration of 0.1 μM at 1 h pre-OGD (Fig. 1c), during OGD (Fig. 1d), or at 1 h post-OGD (Fig. 1e) was evaluated in order to demonstrate the extent of cytoprotection provided by moscatilin against the OGD. The effect of 0.1 μM moscatilin was found to be greatest when administered at 1 h pre-OGD (Fig. 1c), followed by during OGD (Fig. 1d). There was no protective effect at 1 h post-OGD (Fig. 1e).

Cell viability was compared against the DMSO+DMEM group (normal control: 100%; *n* = 6) after OGD and pre-administration of DMSO. Cell viability was significantly (*P* < 0.001) reduced (53.66 ± 2.67%) in the group DMSO+OGD (Fig. 1f). Furthermore, as compared with the DMSO+OGD group, administration of 0.1 μM moscatilin 1 h pre-OGD (Fig. 1f; 62.65 ± 4.35%; *P* = 0.04) resulted in a significant protective effect against the OGD. However, administration of 0.1 μM moscatilin during OGD (Fig. 1f; 56.03 ± 4.08%; *P* = 0.31), or at 1 h post-OGD (Fig. 1f; 52.61 ± 4.16%; *P* = 0.41) did not significantly protect cells against the OGD.

As compared to the DMEM alone (10^5^ cells/ml = 100%), DMSO (50 μl) did not significantly affect cell numbers (DMSO+DMEM, 97.66±1.66%; n = 6). Similarly, moscatilin (0.1 μM) also did not influence cell numbers (moscatilin+DMEM, 99.25 ± 6.05%; *n* = 4). Likewise, compared to DMEM+OGD (53.85±7.03%; n = 4), DMSO (50 μl) did not induce a significant change in cell numbers (DMSO+OGD, 53.66±2.67%; n = 6) as mentioned above.

### The effect of moscatilin on the expression of NDP relative to β-actin in vitro

**Fig. 2:**
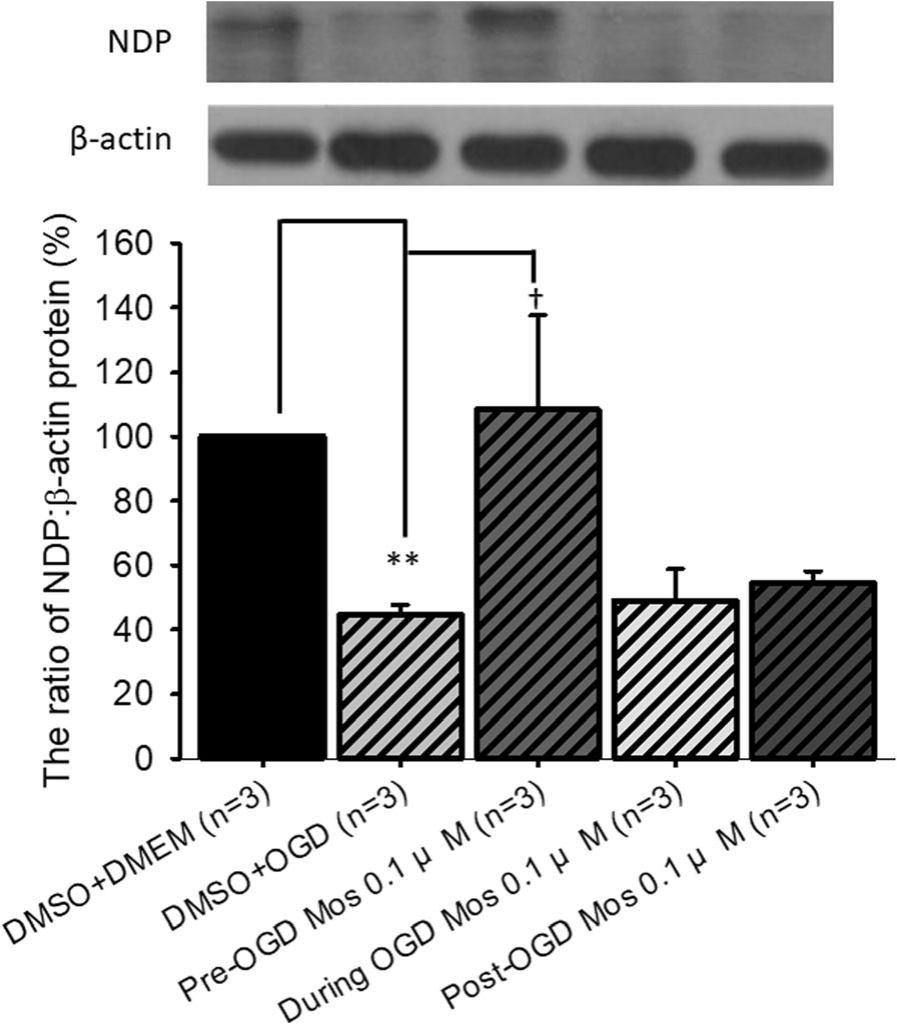
The effect of moscatilin on the protein expression levels of NDP relative to β-actin. Top: a series representative immunoblotting images. Bottom: bar chart. The protein expression level of NDP vs. β-actin in the normal control (DMSO+DMEM: RGC-5 cells cultured in DMEM and pre-administration of DMSO) was adjusted to 100%. ** indicates a significant difference (*P* < 0.01) between the “DMSO+DMEM” group and the “DMSO+OGD” group. † indicates a significant difference (*P* = 0.048) between the “DMSO+OGD” and “Pre-OGD 0.1 μM Mos” groups (0.1 μM moscatilin given 1 h pre-OGD). The results are presented as means±S.E.M. (*n* = 3). Please refer to definitions of various groups in Table 1. Abbreviations: NDP, Norrie disease protein; DMSO, dimethyl sulfoxide; DMEM, Dulbecco’s modified Eagle’s medium; OGD, oxygen glucose deprivation; RGC, retinal ganglion cell

In order to examine alterations in vasculogenesis related NDP, representative immunoblotting images and an analytical bar chart are presented in Fig. 2. When compared to the DMSO+DMEM group (normal control: 100%; *n* = 3), pre-OGD administration of vehicle followed by OGD (DMSO+OGD; *P* < 0.001) significantly reduced the amount of NDP to 44.54 ± 3.15%. When the DMSO+OGD group was compared to the pre-OGD moscatilin+OGD group, there was a significant (*P =* 0.048) increase in the amount of NDP (108.38 ± 29.33%). This elevation in protein expression was greatest when the moscatilin was administered 1 h before the OGD, followed by 1 h post-OGD (54.36 ± 3.88%), with the least effect occurring during OGD (48.99 ± 9.89%).

### The effect of DNL on ERG b-wave

**Fig. 3:**
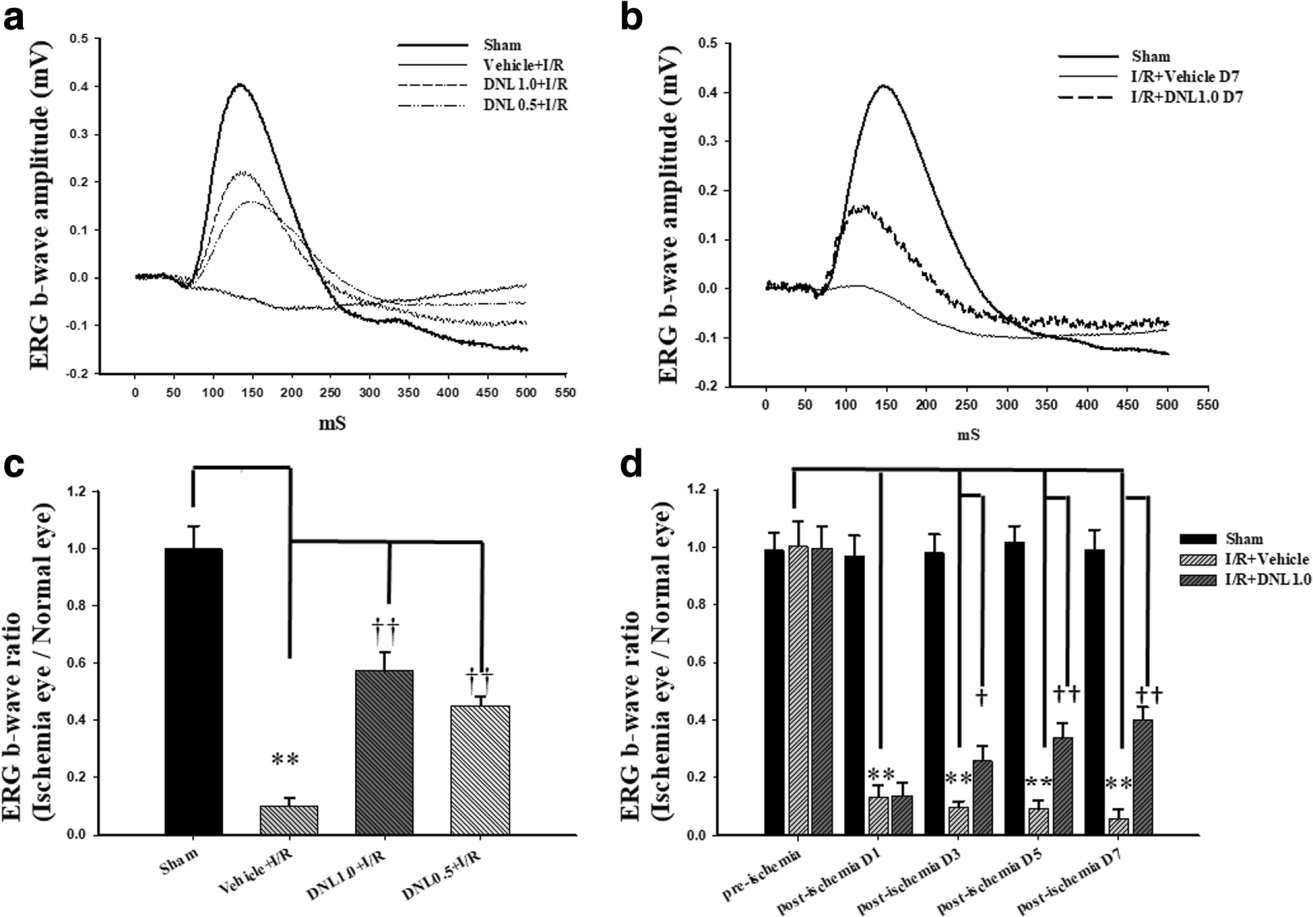
Electroretinogram (ERG) analysis. **a** and **b** Compared to the control retina (Sham), there was a drastic decrease in the ERG b-wave amplitudes after HIOP-induced retinal I/R and pre-administration (**a**) or post-administration (**b**) of vehicle. This decrease was dose-dependently counteracted by pre-ischemia administration of DNL (DNL1.0 + I/R; DNL0.5 + I/R; **a**) or post-ischemia administration of DNL (I/R + DNL1.0; **b**). **c** Compared to the normal control (Sham), a significant (**; *P* < 0.01) decrease in the ERG b-wave ratio occurred in the “Vehicle+I/R” group after retinal I/R. A significant (††; *P* < 0.01) counteraction of this ischemia-induced reduction was dose-responsive and obtained when there was pre-administration of a high dose (DNL1.0 + I/R) and low dose (DNL0.5 + I/R) of DNL. **d** ERG b-wave amplitude was found to be significantly (**; *P* < 0.01) reduced on day 1, 3, 5 or 7 after retinal I/R and post-ischemia administration of vehicle. A significant (†/††; *P* < 0.05/0.01) alleviation of this reduction in ERG b-wave amplitude was achieved by post-administration of DNL (I/R + DNL1.0). Please refer to definitions of various groups in Table 2. Abbreviations: HIOP, high intraocular pressure; I/R, ischemia plus reperfusion. DNL, Dendrobium nobile Lindley. The results are present as means±S.E.M. (*n* = 10~ 12)

We next examined the retinal electrophysiological functioning. After the sham procedure (Sham, Fig. 3), the ERG b-wave amplitude was measured and found to be 0.41 mV. Following retinal ischemia, there was a drastic reduction in b-wave amplitude and this was not affected by either pre-ischemia or post-ischemia treatment with vehicle (Vehicle+I/R: 0.03 mV, Fig. 3a; I/R + Vehicle: 0.07 mV, Fig. 3b). However, pre-ischemia (DNL0.5 + I/R; DNL1.0 + I/R; Fig. 3a) or post-ischemia treatment with DNL (I/R + DNL1.0 D7; Fig. 3b) was able to alleviate the ischemia-induced b-wave decrease, raising the amplitudes for the three groups to 0.18, 0.22 and 0.15 mV, respectively. Furthermore, pre-ischemia treatment with DNL was also found to dose-dependently attenuate the amplitude decrease.

As shown in Fig. 3c (*n* = 12), compared to the Sham group (1.00 ± 0.08), the b-wave ratio in the Vehicle+I/R group (0.10 ± 0.03) was decreased significantly (*P* = 0.002). Importantly, pre-ischemia treatment with DNL dose-responsively and significantly [DNL1.0 + I/R: 0.57 ± 0.06; DNL0.5 + I/R: 0.45 ± 0.03 (*P* < 0.001)] mitigated the ischemia-induced b-wave ratio decrease following I/R.

In Fig. 3d (*n* = 10), compared to the Sham group, the b-wave ratio was significantly (*P* < 0.001) decreased in the I/R + Vehicle group (day 1: 0.13 ± 0.04; day 3: 0.10 ± 0.02; day 5: 0.09 ± 0.03; day 7: 0.06 ± 0.03). Importantly, post-ischemia administration of DNL (I/R + DNL1.0) significantly [D1: 0.14 ± 0.04; D3: 0.26 ± 0.05 (*P* = 0.02); D5: 0.34 ± 0.05 (*P* = 0.003); D7: 0.40 ± 0.04 (*P* < 0.001)] reduced the ischemia-induced b-wave ratio decrease. The pre-ischemia (day 0) b-wave ratios were 1.00 ± 0.09 (I/R + Vehicle) and 1.00 ± 0.08 (I/R + DNL1.0), respectively. When a comparison between the ERG b-wave ratios of the Sham group on days 0, 1, 3, 5 and 7 (0.99 ± 0.06, 0.97 ± 0.07, 0.98 ± 0.06, 1.02 ± 0.01 and 0.99 ± 0.07) was made and they were not significantly different.

### The effect of DNL on the thickness of the retinal layers labeled with cresyl violet

**Fig. 4:**
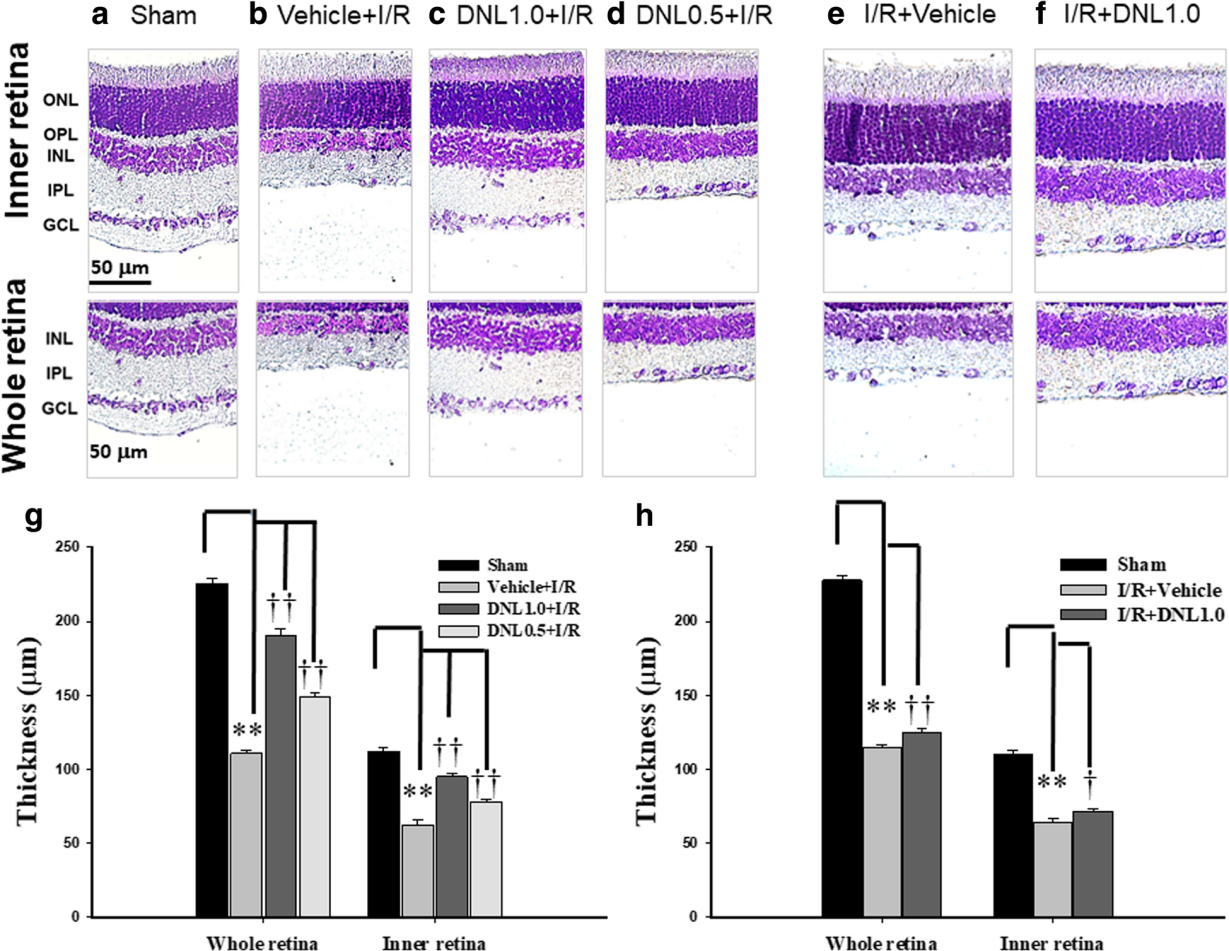
Analysis of the thickness of the whole or inner retina labeled with cresyl violet. **a**, **b**, **e** Shows a retina that received the sham procedure (Sham), or I/R and pre-administration (**b**)/post-administration of vehicle (**e**). **c**, **d**, **f** Are retinas that have undergone I/R and pre-administration of 0.5 g/kg/day (**c**, DNL0.5 + I/R), or 1.0 g/kg/day (**d**, DNL1.0 + I/R), or post-administration of 1.0 g/kg/day (**f**, I/R + DNL1.0) of DNL. The thickness of the whole or inner retina obtained from sections of equal eccentricity that were morphometrically analyzed (**g**, **h**). The results are presented as means±S.E.M. (*n* = 10~ 12). ** represents a significant difference (*P <* 0.01) from the Sham retina. † or †† represents a significant difference (*P <* 0.05 or *P <* 0.01) from the Vehicle+I/R or I/R + Vehicle. Abbreviations: I/R, ischemia plus reperfusion; DNL, Dendrobium nobile Lindley; ONL, outer nuclear layer; OPL, outer plexiform layer; INL, inner nuclear layer; IPL, inner plexiform layer; GCL, ganglion cell layer. Scale bar = 50 μm

Retinal thickness was assessed by sectioning retinal samples at the same distance (1.5 mm) from disc across various groups (n = 10~ 12; Fig. 4). Compared to retinas that had received the sham procedure (Sham, Figs. 4a and g: 225.50 ± 3.26 μm for the whole retina, 112.08 ± 2.58 μm for the inner retina), the retinal thicknesses of the animal pre-administrated with vehicle and subjected to I/R (Vehicle+I/R, Fig. 4b and g: 110.83 ± 1.85 μm for the whole retina, 62.50 ± 3.06 μm for the inner retina) were significantly (*P* < 0.001) decreased. Moreover, this decrease was dose-dependently and significantly (*P* < 0.001) counteracted when the animal received I/R and pre-administration of DNL (DNL1.0 + I/R, Fig. 4c and g: 190.08 ± 4.48 μm for the whole retina, 94.92 ± 2.27 μm for the inner retina; DNL0.5 + I/R, Fig. 4d and g: 148.58 ± 2.80 μm for the whole retina, 78.25 ± 1.53 μm for the inner retina).

In contrast to retinas subjected to the sham procedure (Sham, Fig. 4a), the retinal thicknesses of the rats that were given I/R and post-administration of vehicle (I/R + Vehicle, Fig. 4e and h: 115.00 ± 2.04 μm for the whole retina, 63.92 ± 3.30 μm for the inner retina) were significantly (*P* < 0.001) reduced. Moreover, post-administration of DNL blunted this ischemia-induced reduction significantly [I/R + DNL1.0; Fig. 4f and h: 125.25 ± 2.66 μm for the whole retina (*P* = 0.006); 71.50 ± 1.51 μm for the inner retina (*P* = 0.048)].

### The effect of DNL on the density of retrograde fluorogold immunolabeled RGCs

**Fig. 5:**
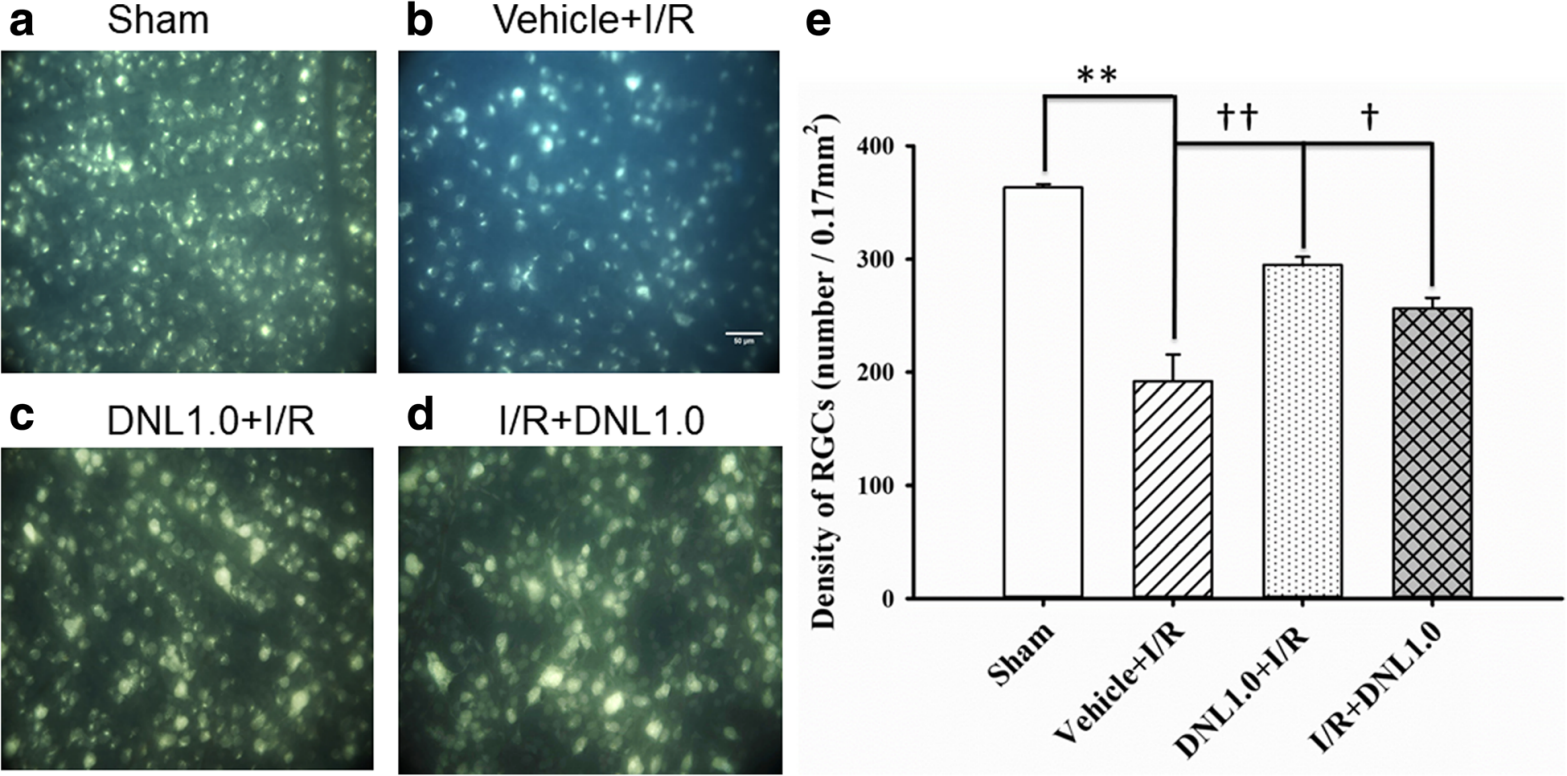
Fluorogold labeling. The micrographs shows the retinal ganglion cell (RGC) density after the sham procedure (**a**, Sham), or after ischemia followed by reperfusion (I/R) plus pre-ischemia administration of vehicle (**b**, Vehicle+I/R) or pre−/post-ischemia administration of DNL at 1.0 g/kg/day (**c**, DNL1.0 + I/R; **d**, I/R + DNL1.0). The RGC density was quantitatively analyzed (**e**). As indicated by each bar, the results were means±S.EM. (*n* = 4). ** indicates a significant difference from the sham retina (*P* < 0.01; Sham vs. Vehicle+I/R); †† or † indicates a significant difference from the Vehicle+I/R (*P* < 0.01 or *P* < 0.05; Vehicle+I/R vs. DNL1.0 + I/R or I/R + DNL1.0). DNL, Dendrobium nobile Lindley. Scale bars = 50 μm

When RGC density was assessed (Fig. 5; *n* = 4), the density of the sham group (Sham, Figs. 5a and e) was 363.23 ± 2.84 cells/field. Compared to the Sham group, there was a significant (*P* < 0.001) reduction in RGC density (192.06 ± 23.53 cells/field) in animals that underwent retinal ischemia and pre-administration of vehicle (Vehicle+I/R, Figs. 5b and e). Furthermore, this decrease was significantly (*P* = 0.006 or 0.045) mitigated when the animals received either retinal ischemia and pre-administration of DNL (DNL1.0 + I/R; Figs. 5c and e: 295.15 ± 7.14 cells/field), or post-ischemia administration of DNL (I/R + DNL1.0; Figs. 5d and e: 256.26 ± 9.46 cells/field).

### The effect of DNL efficacy on ChAT immunoreactivity

**Fig. 6:**
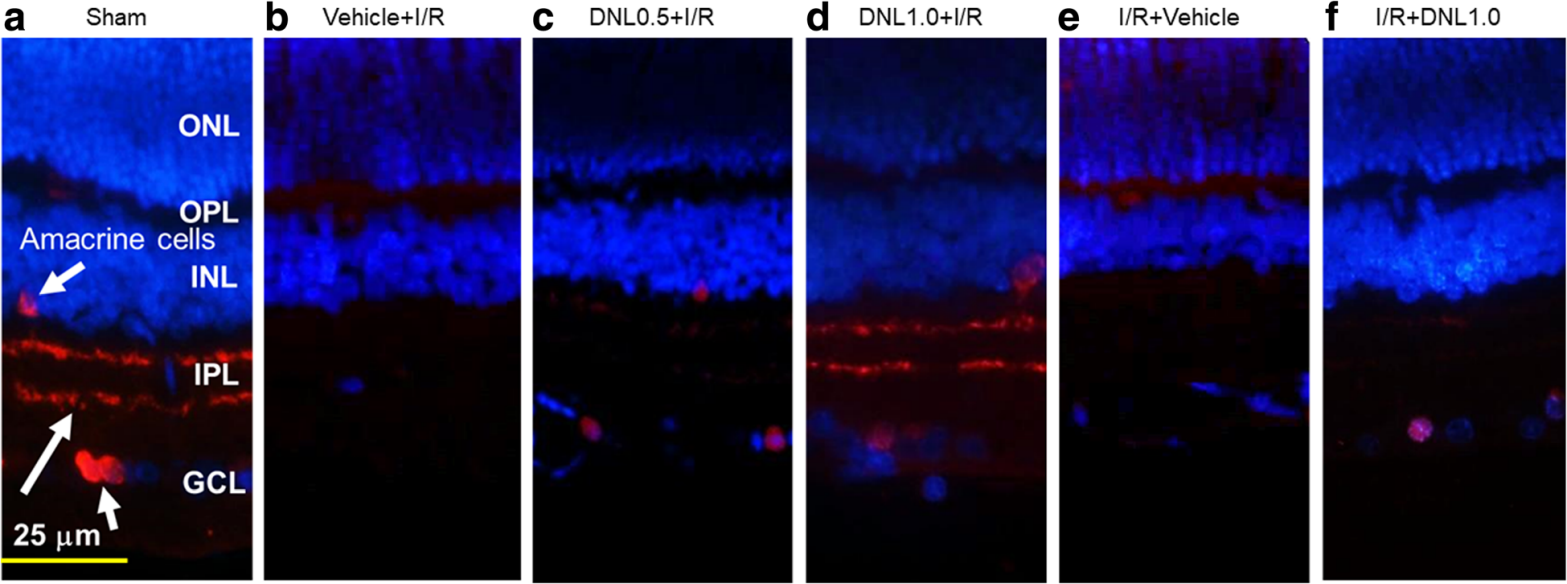
Choline acetyltransferase (ChAT, red) immunohistochemical studies. **a** Shows a retina that have undergone the sham procedure (Sham); cell nuclei are counterstained with 4,6-diamidine-2-phenylindole dihydrochloride (DAPI, blue). Amacrine cell bodies (Sham; short arrows) can be seen in the INL and GCL and their neuronal processes (long arrow) display a two-band pattern in the IPL. **b**, **e** Show retinas that have undergone I/R together with pre−/post-administration of vehicle (Vehicle+I/R or I/R + Vehicle). A considerable reduction in the IPL immunoreactivity can be seen together with a great reduction in the number of amacrine cell bodies. **c**, **d**, **f** show sectioned retinas that have received I/R and pre-administration of 0.5 g/kg/day (c, DNL0.5 + I/R), or 1.0 g/kg/day of DNL (d, DNL1.0 + I/R), or that have received I/R and post-administration of 1.0 g/kg/day of DNL (f, I/R + DNL1.0). In these groups, the ischemia-induced changes can be seen to be obviously and dose-dependently mitigated when the ischemic retinas have received pre-administration of 0.5 and 1.0 g/Kg/day of DNL. Post-administration of 1.0 g/Kg/day of DNL can also be seen to obviously mitigate these ischemia-induced changes. Abbreviations: ONL, outer nuclear layer, OPL, outer plexiform layer, INL, inner nuclear layer, IPL, inner plexiform layer, GCL, ganglion cell layer. DNL, Dendrobium nobile Lindley. Scale bar = 25 μm

ChAT immunoreactivity in the retina after the sham procedure (Sham; Fig. 6a) is able to pinpoint ChAT (red) immunolabeling of the amacrine cell bodies (short arrows) present in the INL and ganglion cell layer (GCL). This procedure also demonstrates the presence of two distinct strata (long arrow) within the inner plexiform layer (IPL). In retinas that had undergone ischemia and pre−/post-administration of vehicle (Vehicle+I/R; Fig. 6b; I/R + Vehicle, Fig. 6e), the numbers of CHAT-immunolabeled amacrine cell bodies were drastically decreased; furthermore, their IPL immunolabeling was considerably reduced. It is clinically important to note that these changes were nullified in a dose-dependent manner when the ischemic retinas had received pre-administration of DNL (DNL0.5 + I/R, Fig. 6c; DNL1.0 + I/R, Fig. 6d). Additionally, post-administration of DNL (I/R + DNL1.0, Fig. 6f) also obviously mitigated these ischemia-induced changes. Merge images of ChAT immunolabeling and DAPI staining of cellular nuclei are used for all presented pictures.

### The effect of DNL on vimentin and GFAP immunoreactivity

Immunohistochemical investigations were carried out with the aim of investigating the vimentin immunoreactivity and GFAP immunoreactivity.

#### Vimentin immunohistochemistry

**Fig. 7:**
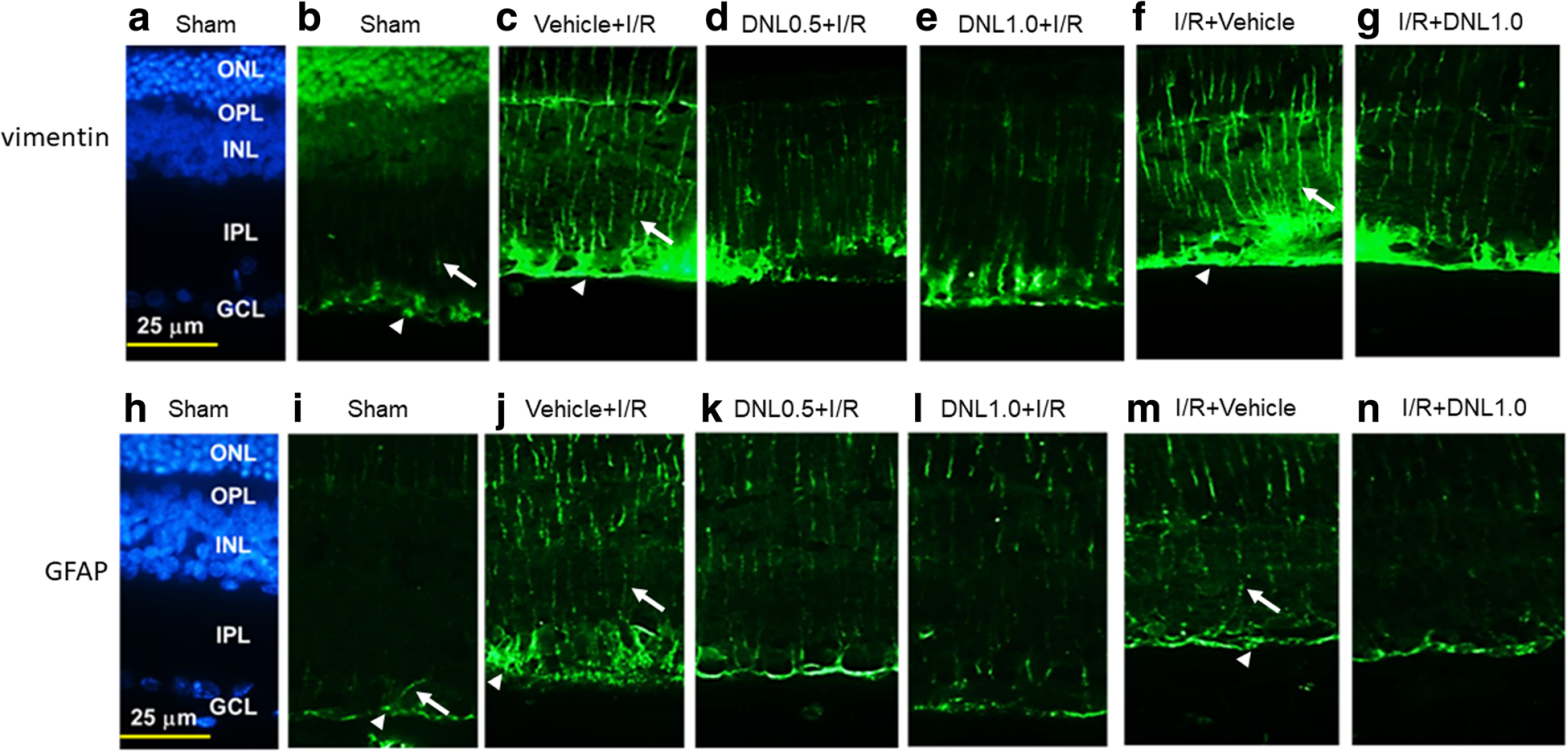
Vimentin immunohistochemistry. **b** After the sham procedure (Sham), anti-vimentin (green) immunoreactivity can be seen in the end feet of Müller cells (arrow heads; see also **c** and **f**) within the ganglion cell layer (GCL) as well as their processes are also immunolabeled in the IPL (arrows; see also c and f), INL and ONL. **c**, **f** Compared to the sham retina, there was a considerable enhancement of the anti-vimentin immunolabeling after ischemia and pre-administration or post-administration of vehicle (Vehicle+I/R or I/R + Vehicle). **d**, **e**, **g** This enhancement was obviously counteracted by pre-administration of 0.5 g/kg/day (DNL0.5 + I/R), or 1 g/kg/day of DNL (DNL1.0 + I/R), or post-administration of 1 g/kg/day of DNL (I/R + DNL1.0). GFAP immunohistochemical study. After the sham procedure (Sham; **i**), the Müller cells displayed GFAP immunoreactivity at their end feet within the GCL (arrow heads; see also **j** and **m**), and at their processes in the IPL (arrows; see also **j** and **m**), INL and ONL. Compared to the Sham retina, anti-GFAP immunolabeling was enhanced after ischemia and pre-administration/post-administration of vehicle (Vehicle+I/R, **j**; I/R + Vehicle, **m**). This enhancement was mitigated by pre-administration of 0.5 g/kg/day (DNL0.5 + I/R; **k**) or 1 g/kg/day of DNL (DNL1.0 + I/R; **l**), or post-administration of 1 g/kg/day of DNL (I/R + DNL1.0; **n**). **a**, **h** DAPI (blue) was used to counterstain cell nuclei in the sham retina. DNL, Dendrobium nobile Lindley. GFAP, glial fibrillary acidic protein; IPL, inner plexiform layer; INL, inner nuclear layer; ONL, outer nuclear layer; DAPI, 4,6-diamidine-2-phenylindole dihydrochloride. Scale bar = 25 μm

In the control retina (Sham, Fig. 7b), the Müller cell processes showed vimentin immunolabeling at the end feet (arrow heads; see also Figs. 7c and f) in the GCL as well as at the processes that extended into the IPL (arrows; see also Figs. 7c and f), INL and ONL. Compared to the control retina (Sham, Fig. 7b), an increase in the anti-vimentin immunoreactivity was found after retinal I/R and pre−/post-administration of vehicle (Vehicle+I/R, Fig. 7c; I/R + Vehicle, Fig. 7f). This increase was considerably and dose-dependently blunted by pre-administration of DNL (DNL0.5 + I/R, Fig. 7d; DNL1.0 + I/R, Fig. 7e). Furthermore, post-administration of DNL (I/R + DNL1.0, Fig. 7g) also drastically nullified this ischemia-induced change.

#### GFAP immunohistochemistry

Compared to the control retina (Sham, Fig. 7i), an increase of the anti-GFAP immunolabeling was observed in the ischemic retina pre/post-administrated with vehicle (Vehicle+I/R, Fig. 7j; I/R + Vehicle, Fig. 7m). Moreover, this change was obviously and dose-responsively reduced when the ischemic retinas were preadministered with DNL (DNL0.5 + I/R, Fig. 7k; DNL1.0 + I/R, Fig. 7l). Post-administration of DNL (I/R + DNL1.0, Fig. 7n) also obviously reduced this ischemia-induced change. DAPI (blue; Figs. 7a and h) was used to stain cellular nuclei of the Sham retina.

### The effects of DNL on the levels of various proteins in the rat retina

**Fig. 8:**
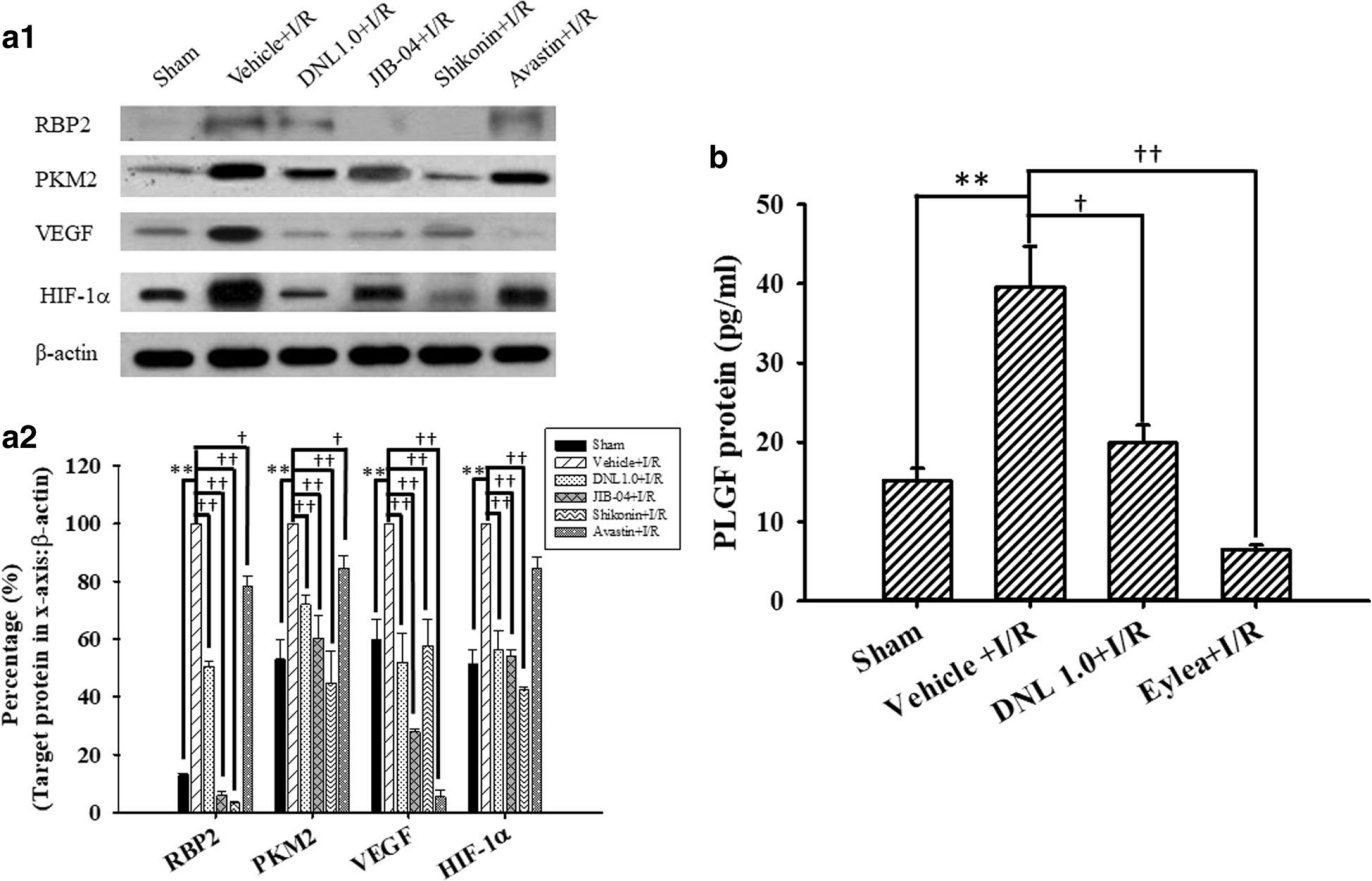
Western blot analysis showing the expression levels of β-actin, HIF-1α, VEGF, PKM2 and RBP2. Lane 1 or 2 of picture **a1** shows a sham retina subjected to the sham procedure (Sham), or a ischemic retina pre-administered with vehicle (Vehicle+I/R), respectively. Lane 3 shows a retina that received ischemia together with pre-ischemia administration of 1 g/kg/day DNL (DNL1.0 + I/R). Lanes 4~ 6 show retinas that underwent ischemia together with pre-ischemia administration of 10 μM/5 μl JIB-04 (RBP2 inhibitor), 4 μM/5 μl shikonin (PKM2 inhibitor), or 125 μg/5 μl avastin (anti-VEGF), respectively. Each bar in picture **a2** represented the ratio of RBP2, PKM2, VEGF and HIF-1α to β-actin. ** indicates a significant (*P* < 0.01) difference between the Sham retina and the ischemic retina pre-administered with vehicle (Vehicle+I/R). † or †† indicated a significant (*P* < 0.05 or *P* < 0.01) difference between the “Vehicle+I/R” group and the ischemic retina pre-administered with DNL1.0, JIB-04, shikonin or avastin. The results are presented as means±S.E.M. (*n* = 4~ 10). The results of the ELISA assay are presented in picture **b**. The concentration of PLGF was measured in the retinas obtained from various groups, namely Sham, Vehicle+I/R, DNL1.0 + I/R or Eylea+I/R. Rats that received I/R plus pre-ischemia administration of 200 μg/5 μl Eylea was defined as the “Eylea+I/R” group. ** indicates a significant difference (*P <* 0.01) between the Sham retina and Vehicle+I/R. † or †† indicated a significant (*P* < 0.05 or *P* < 0.01) difference between Vehicle+I/R and DNL1.0 + I/R or between Vehicle+I/R and Eylea+I/R. Results are presented as means±S.E.M. (n = 4). Abbreviations: DNL, Dendrobium nobile Lindley. HIF-1α, hypoxia inducible factor-1α; VEGF, vascular endothelium factor; PKM2: pyruvate kinase M2, RBP2: retinoblastoma-binding protein 2; PLGF, placental growth factor

The levels of various proteins in the control retinas (Sham; Table 3; *n* = 4~ 10) were measured and the results are shown in Fig. 8a1 and a2 (HIF-1α = 51.17 ± 5.14%; VEGF = 59.72 ± 6.94%; PKM2 = 52.93 ± 7.01%; RBP2 = 12.81 ± 0.55%). After I/R and preadministration of vehicle, significant (all *P* ≤ 0.001) elevations were observed in the levels of HIF-1α, VEGF, PKM2 and RBP2 (normalized to 100%). Furthermore, these elevations were significantly (all *P* < 0.001; HIF-1α = 56.08 ± 6.76; VEGF = 51.87 ± 9.89; PKM2 = 71.99 ± 3.05; RBP2 = 50.64 ± 1.48) inhibited when the ischemic retinas were preadministered with 1.0 g/Kg/day of DNL. Additionally, there was a significant (*P* ≤ 0.002 except those for avastin) attenuation of the ischemia-induced increase in the levels of HIF-1α [JIB-04 = 53.98 ± 2.29; shikonin = 42.65 ± 0.76; avastin = 84.61 ± 3.96 (*P* = 0.07)], VEGF (JIB-04 = 27.82 ± 1.21; shikonin = 57.55 ± 9.40; avastin = 5.38 ± 2.51), PKM2 [JIB-04 = 60.36 ± 7.59; shikonin = 44.94 ± 10.91; avastin = 84.44 ± 4.53 (*P* = 0.01)], and RBP2 (JIB-04 = 5.83 ± 1.43; shikonin = 3.40 ± 0.23; avastin = 78.35 ± 3.29 (*P* = 0.02)] after pre-administration of each inhibitor/antibody JIB-04 (RBP2 inhibitor), shikonin (PKM2 inhibitor) and avastin (VEGF antibody).

As shown in Fig. 8b (n = 4), in contrast to the control retinas (Sham = 15.11 ± 1.58 pg/ml), after I/R and preadministration of vehicle, there was a significant (*P* = 0.004) elevation in the level of PLGF (Vehicle+I/*R* = 39.53 ± 5.25). Moreover, this elevation was significantly (*P* = 0.01 or *P* < 0.001) blunted when the ischemic retinas were pre-administrated with DNL (DNL1.0 + I/*R* = 19.93 ± 2.24), or anti-PLGF antibody Eylea (Eylea+I/*R* = 6.44 ± 0.60).

## Discussion

As mentioned in the Introduction, many of DNL’s various active components, including alkaloids, flavonal glycosides, SG-168 and polysaccharides, have known action mechanisms [18–21]; on the other hand, moscatilin is also an active ingredient (bibenzyl) of DNL seems to have a novel mode of action. In the present study, we found that the protein levels of HIF-1α, VEGF, PKM2 and RBP2 were significantly upregulated in the ischemic retinas, which agrees with previous studies [8, 10–12], but importantly the significant upregulation events affecting these proteins were significantly mitigated by administration of DNL; furthermore, this did not happen with vehicle alone (Fig. 8a). As shown in Fig. 8b, the ischemia-induced elevation of PLGF [13] was also significantly blunted when ischemic retinas were pre-administrated with the VEGF trap/anti-PLGF Eylea. This is similar to the effect of 1.0 g/Kg/day of DNL. This finding suggests that DNL might have a novel and clinical significant anti-angiogenesis/VEGF (PLGF) trapping effect. This is not inconsistent with previous reports where a bibenzyl component of DNL, moscatilin, has been shown to act as an anti-angiogenesis agent and inhibit HIF-1α and VEGF [15, 16, 22].

Up to the present, it has been believed that ischemia is likely to be very similar in mode of action to various diseases such as CRVO/BRVO/CRAO/BRAO, nvAMD and DR, as well as various developmental retinal vasculopathies such as familial exudative vitreoretinopathy and Norrie disease. Over the past twenty years, anti-VEGF antibodies have been used to clear ocular hemorrhage and macular edema effectively in many cases; however, disappointingly, poor visual results do occur in some patients. An increasing body of evidence supports a role of the norrin-dependent Wnt-signaling pathway in both the early normal development of retinal vessels and in the late progression of defined developmental retinal vascular diseases [1–5, 7]. The latter condition may possibly further aggravate ischemia/hypoxia and form NV. Consistently, NDP (norrin) seems to protect the eye from abnormal angiogenesis and retinopathy and it does this by modulating the norrin-dependent Wnt signaling pathway [30]. Moreover, overexpression of NDP has been shown to protect photoreceptors and RGCs from cell death via activation of the norrin-dependent Wnt signaling pathway [31, 32]. Presently, hypoxia (OGD) is known to lead to a significant decrease in the level of NDP (Fig. 2) as well as to cause a significant decrease in the cell viability (Fig. 1). Furthermore, a significant nullification in the OGD induced reduction in the NDP level (Fig. 2) and cultured cell number (Fig. 1) in the presence of moscatilin (0.1 μM), implies that this bibenzyl ingredient of DNL is to be able to significantly alleviate hypoxic/ischemic-like (OGD) injury. As supported by the present results and by various previous reports [5, 7, 15, 16, 22, 30–32], DNL and/or moscatillin would seem to be able to activate the NDP (norrin)-dependent Wnt signaling pathway (Graphical Abstract for DNL & moscatilin as anti-PLGF & NDP stimulator; Additional file 1) and thus provide neuroprotection against retina ischemia (Figs. 1, 3, 4, 5, 6 and 7). This presumably occurs via an inhibition of VEGF-A/PLGF (Fig. 8) and an upregulation of NDP (Fig. 2). This approach seems to be a novel promising way of protecting against retinal ischemia that should further terminate abnormal vasculogenesis, ischemia associated neovascularization (angiogenesis), and the progression of various developmental vascular disorders that are associated with persistent ischemia/hypoxia [5, 7].

After ischemic insult and the administration of vehicle, the inner retinal thickness (Fig. 4), the number of RGCs (Fig. 5) and the ChAT immunoreactivity of amacrine cells (Fig. 6) were significantly/obviously decreased, which is not inconsistent with a previous report [8]. Importantly, our findings in the present study also confirm that these ischemia induced changes were significantly or obviously blunted by pre- administration or post-administration of a high dose of DNL (at 1 g/kg/day). Moreover, in the ischemic retinas with pre−/post-administration of vehicle, vimentin/GFAP immunolabeling overexpression (Fig. 7) was found to parallel the decrease in b-wave (Fig. 3). This is of clinical importance because the present results also show that these ischemic alterations are significantly or obviously counteracted by pre-administration or post-administration of DNL at 1 g/kg/day.

The present results demonstrate that ischemia/hypoxia (ischemia-mimetic OGD), significantly or obviously affects the retina electrophysiologically (Fig. 3), morphometrically (Fig. 4), and immunohistochemically (Figs. 5, 6 and 7) as well as affect at the level of molecular biology and cellular viability (Figs. 1, 2 and 8). In terms of clinical situation, all of these changes following ischemia/OGD are effectively attenuated by pre-treatment or post-treatment with DNL or its bibenzyl component moscatilin (Figs. 1, 2, 3, 4, 5, 6, 7 and 8). This is the first study to show that the Chinese herb DNL (and its bibenzyl ingredient moscatilin) [22, 33] might be able to electrophysiologically, morphometrically, immunohistochemically and molecular biologically protect against the retinal ischemic/ischemic-like injury. These protective mechanisms are presumed to act by suppressing the upregulation of HIF-1α, VEGF-A, PKM2, RBP2, and, above all, PLGF, as well as, possibly, by upregulating the level of NDP (Figs. 2 and 8).

Taking the above findings as a whole, DNL and/or moscatilin seems to be able to protect against or even prevent defined retinal ischemic/ischemic-like alterations and this is likely to occur via the inhibition of the PLGF and also probably via the upregulation of NDP. DNL treatment (and/or moscatilin) might be an useful way of providing a complementary approach that helps to prevent and/or manage the developmental vascular disorders such as Norrie disease in patients where the diseases might progress due to the presence of persistent ischemia/hypoxia [5, 7].

## Conclusions

The present study has that demonstrated various ischemic/hypoxic (OGD) alterations that occur in the retina or in retinal cells and these can be monitored by electroretinography, immunohistochemistry (RGCs, amacrine cells, Müller cells), histopathology (retinal thickness), cell viability and measurement of expression levels of various proteins (PLGF, HIF-1α, VEGF-A, PKM2, RBP2 and NDP). Of clinical significance and novel to this study, the protein concentration of PLGF (Fig. 8) was found to be upregulated in such circumstances and that of NDP (Fig. 2) was downregulated. Moreover, and importantly, treatment with DNL/moscatillin (DNL’s bibenzyl ingredient) significantly counteracted these alterations. Furthermore, neither moscatilin nor DMSO has an effect on cell numbers in the control groups [moscatilin (0.1 μM) + DMEM or DMSO (50 μl) + DMEM]. DNL/moscatillin might safely provide an alternative way to prevent and/or manage patients with the persistent hypoxia/ischemia associated progression that occurs in various developmental vascular disorders such as Norrie disease; this might occur via a downregulation of the level of PLGF and an upregulation of the concentration of NDP.

## Additional file

Additional file 1: DNL & moscatilin protect retinal cells from ischemia/hypoxia by dowregulating PLGF and upregulating NDP. (TIF 173 kb)

## Abbreviations

AMD: Age-related macular degeneration
ANOVA: Analysis of variance
ARVO: Association for research in vision and ophthalmology
BCA: Bicinchoninic acid
BRAO: Branch retinal artery occlusion
BRVO: Branch retinal vein occlusion
BSA: Bovine serum albumin
ChAT: Choline acetyltransferase
CHGH: Cheng Hsin General Hospital
CHIACUC: Institutional Animal Care and Use Committee at CHGH
CRAO: Central retinal artery occlusion
CRVO: Central retinal vein occlusion
DAPI: 4,6-diamidine-2-phenylindole dihydrochloride
DMEM: Dulbecco’s modified eagle’s medium
DMSO: Dimethyl sulfoxide
DNL: Dendrobium nobile Lindley
ECL: Enhanced chemiluminescent
ERG: Electroretinogram
FEVR: Familial exudative vitreoretinopathy
FITC: Fluorescein isothiocyanate
FITC: Fluorescein isothiocyanate
Fzd-4: Frizzled-4
GCL: Ganglion cell layer
GFAP: Glial fibrillary acidic protein
HIF-1α: Hypoxia-inducible factor-1α
HIOP: High intraocular pressure
HRP: Horseradish peroxidase
I/R: Ischemia/reperfusion
ILM: Internal limiting membrane
INL: Inner nuclear layer
IPL: Inner plexiform layer
MPER: Mammalian protein extraction reagent
MTT: 3-(4,5-dimethylthiazol-2-yl)-2,5-diphenyltetrazolium bromide
NADPH: Nicotinamide adenine dinucleotide phosphate hydrogen
NDP: Norrie disease protein
NV: Neovascularization
OD: Optical density
OGD: Oxygen glucose deprivation
PBS: Phosphate-buffered saline
PBST: Phosphate buffered saline containing 0.05% Tween 20
PDR: Proliferative diabetic retinopathy
PHPV: Persistent hyperplastic primary vitreous disease
PKM2: Pyruvate kinase M2
PLGF: Placental growth factor
PVDF: Polyvinylidene difluoride
RBP2: Retinoblastoma-binding protein 2
RGCs: Retinal ganglion cells
RPE: Retinal pigment epithelium
SDS-PAGE: Sodium dodecyl sulfate polyacrylamide gel electrophoresis
SE: Standard error
TMB: Tetramethylbenzidine
VEGF: Vascular endothelium growth factor
