# Supplementary figures and images for: Dendrobium nobile Lindley and its bibenzyl component moscatilin are able to protect retinal cells from ischemia/hypoxia by dowregulating placental growth factor and upregulating Norrie disease protein
Source: BMC Complement Altern Med. 2018 Jun 22;18:193. doi: 10.1186/s12906-018-2256-z (PMC6013934; doi:10.1186/s12906-018-2256-z)

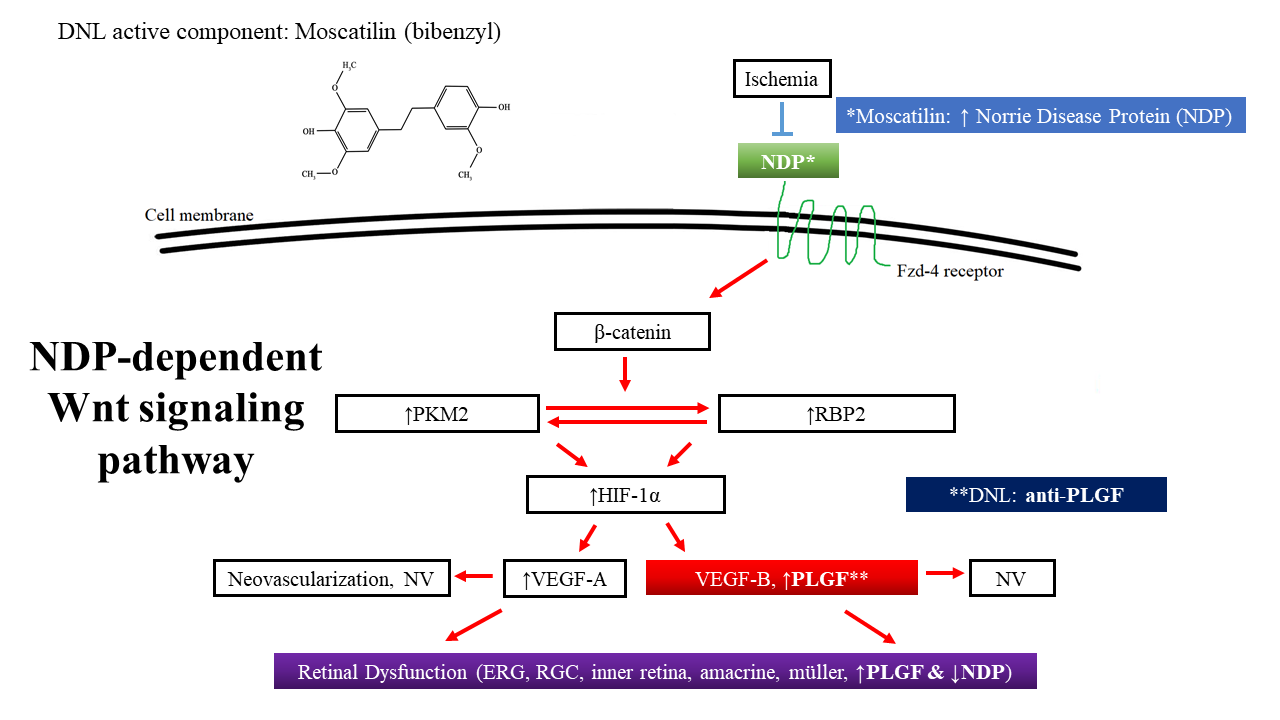

Supplement: Supplementary file 1 — DNL & moscatilin protect retinal cells from ischemia/hypoxia by dowregulating PLGF and upregulating NDP. (TIF 173 kb) [file 12906_2018_2256_MOESM1_ESM.tif]
